# Degradation Mechanism of AAA+ Proteases and Regulation of *Streptomyces* Metabolism

**DOI:** 10.3390/biom12121848

**Published:** 2022-12-10

**Authors:** Weifeng Xu, Wenli Gao, Qingting Bu, Yongquan Li

**Affiliations:** 1 Institute of Pharmaceutical Biotechnology, Zhejiang University School of Medicine, Hangzhou 310058, China; 2 Zhejiang Provincial Key Laboratory for Microbial Biochemistry and Metabolic Engineering, Hangzhou 310058, China

**Keywords:** protein degradation, *Streptomyces*, regulatory network, Lon, FtsH, ClpP, proteasome

## Abstract

Hundreds of proteins work together in microorganisms to coordinate and control normal activity in cells. Their degradation is not only the last step in the cell’s lifespan but also the starting point for its recycling. In recent years, protein degradation has been extensively studied in both eukaryotic and prokaryotic organisms. Understanding the degradation process is essential for revealing the complex regulatory network in microorganisms, as well as further artificial reconstructions and applications. This review will discuss several studies on protein quality-control family members Lon, FtsH, ClpP, the proteasome in *Streptomyces,* and a few classical model organisms, mainly focusing on their structure, recognition mechanisms, and metabolic influences.

## 1. Introduction

Protein homeostasis is an important prerequisite for bacteria to maintain normal activity. In a complex natural living environment, bacteria must deal with external stimuli, such as pressure, pH, temperature, and nutrient supply, and promptly remove the misfolded proteins caused by these factors. On the other hand, bacteria change the expression level of their proteins according to the needs of their own growth. They contain a simpler protein degradation system than eukaryotes. Usually, prokaryotes rely on four cytoplasmic ATP-dependent proteases—Lon, FtsH, Clp, and HslUV—while *Streptomyces* mainly contains the first three proteases. These proteases are part of the AAA+ protein family (ATPases associated with various cellular activities) [1]. Although they play a degrading function by recognizing different substrates, they have a similar structure and are composed of two parts, a peptidase (protease) and an unfoldase (ATPase) [1,2]. ATP hydrolysis by the unfoldase promotes the unfolding and translocation of the substrate protein into peptidase through the axial pore of the entire protease complex. Finally, the unfolded substrate is transferred to the proteolysis chamber, where peptide bond cleavage occurs [3]. In addition, proteins in bacteria can be specifically degraded through the proteasome. The proteasome was first discovered in actinomycetes [4], and then it was extensively studied in *Mycobacterium tuberculosis*, where the important prokaryotic ubiquitin-like protein (Pup) was discovered [5]. Pup can be covalently labeled in a variety of functional proteins; therefore, the Pup-proteasome degradation pathway provides a reliable theoretical basis for the study of bacteria protein degradation.

*Streptomyces* is an indispensable member of the bacterial family, and its secondary metabolite library greatly enriches human clinical drug selection. This library includes antibiotics (such as daptomycin), immunosuppressants (such as rapamycin), antifungal drugs (such as amphotericin B), anticancer drugs (such as adriamycin), and antiparasitic drugs (such as ivermectin), etc., (the compounds described in this review are listed in Table 1) [6]. Their biosynthesis process is tightly controlled by regulatory pathways. In order to obtain more valuable metabolites, researchers have made lots of efforts to study the regulatory network, as well as gene cluster mining [7]. In recent years, the development of proteomics based on high-resolution mass spectrometry (MS) quantitatively revealed acylation types and modification sites. Characterizing the activities of key regulators in microorganisms is important to understand their function. Some researchers found that changes in protein activity could affect the biosynthesis process of *Streptomyces* [8]. Degradation is particularly a significant part of a protein’s lifespan. For the sake of convenience for follow-up research, we present a brief review that uses different types of proteases discussed in different points, combined with other model microorganisms, especially the protease function mechanism in *Streptomyces*.

## 2. Lon

**Figure 1:**
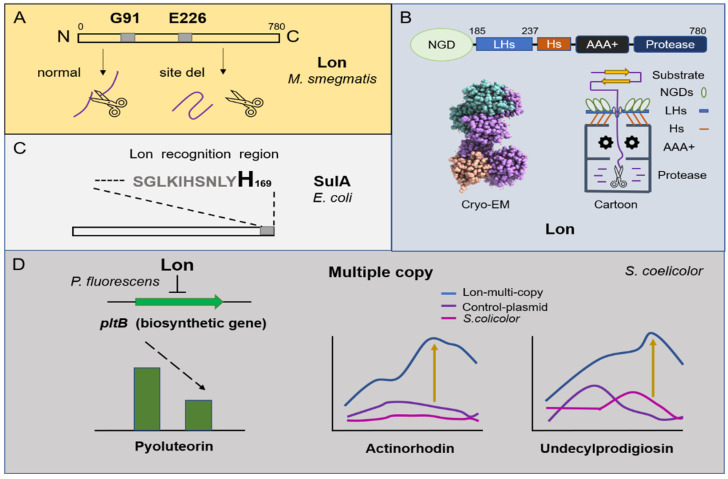
Overview of the degradation mechanism of Lon protease and its regulation on metabolism. (**A**) N-terminal amino acids G91 and E226 of Lon protease from *Mycobacterium smegmatis* are the most important active sites. (**B**) The cartoon structure of Lon protease and the Cryo–EM structure of the N-terminal domain of Lon *E. coli* (The Cryo–EM structure was downloaded from the PDB database). The whole length of Lon contains N-terminal globular domains (NGDs), three-helix bundles (3Hs), interlocked long helices (LHs), an AAA+ domain, and a protease chamber. (**C**) The degradation of SulA by Lon protease depends on its C-terminal histidine. (**D**) Lon protease negatively regulates the production of pyoluteorin in *Pseudomonas fluorescens* Pf-5. The multiple copies of the *lon* gene in *S. coelicolor* cause an increase in the yield of undecylprodigiosin and actinorhodin.

Lon, as a member of the protein quality control family, was identified for the first time in *Escherichia coli* [9]. Early studies indicated that its C-terminal domain in *E. coli* contains a proteolytic active site formed by a serine-lysine catalytic dimer [10] and may be involved in substrate recognition and binding [11,12]. Additionally, in further studies, researchers found that Lon’s N-terminal region also has a certain effect on degradation function. The deletion of N-terminal amino acids G91 and E226 in the Lon protease from *Mycobacterium smegmatis* both severely impaired the degradation of substrates α-casein in vitro and RcsA in vivo (Figure 1A) [13]. The full-length structure of Lon has always been a mystery. Until recently, it was analyzed by cryo–electron microscopy, which revealed a multilayered architecture featuring a tensegrity triangle complex, uniquely constructed by six long N-terminal helices (Figure 1B). However, the details of this are not described here [14]. In addition to its own structure region, a certain amino acid of Lon’s substrate is also closely related to degradation. For example, SulA, which functions in the stage of DNA damage, is a cell differentiation inhibitor and is degraded by the Lon protease in *E. coli.* Its affinity with Lon changes with the mutation of C-terminal histidine (Figure 1C) [15]. SoxS and MarA act as regulators of the redox process and antibiotic resistance enhancement in *E. coli*, respectively, and their half-life is prolonged due to the deletion of Lon. Moreover, inserting histidine at its N-terminus also increases Lon’s stability, suggesting that it may recognize the N-terminal domains of SoxS and MarA and play a role in degradation [16]. Nevertheless, more work is needed to further define the conserved sequences for Lon-mediated degradation.

Lon participates in different aspects of bacterial physiology, including cell differentiation, sporulation, pathogenicity, and survival under starvation conditions, and it can respond to external pressure stimuli. Studies show that NaCl (450 mM) can induce the expression of Lon and that ectoine treatment further enhances the expression level (5.2-fold) in *Streptomyces* sp., strain C-2012 [17]. The ATP-dependent Lon protease is also a heat shock protein. In *Streptomyces coelicolor*, the deletion of the DnaK chaperone machine in vivo results in negative feedback regulation in *lon* and *clpB* [18]. Subsequently, transcriptomics and translatomics showed that Lon and heat shock molecular chaperones (DnaK and GroES/GroEL1/GroEL2) are synergistically upregulated under heat stimulation [19]. In *Streptomyces lividans*, it is confirmed that Lon is a new stress response regulator and belongs to HAIR (HspR co-recognition sequence (CTTGAGT-N7-ACTCAAG)/HspR (heat shock) protein regulator). Briefly, its molecular chaperone interacts with unfolded proteins under heat induction, causing Lon (HspR) to function in a free form for derepression, which suggests that excessive production of Lon protease may be toxic to the bacteria [20,21]. Interestingly, the effect of Lon protease on microorganism metabolic function is relative. On the one hand, studies found that the mutation of Lon could damage spore germination under high temperatures, and Lon negatively regulated the antibiotic production of pyoluteorin in *Pseudomonas fluorescens* Pf-5 by influencing *pltB* biosynthetic gene transcription [22]. On the other hand, after inserting multiple copies of the *lon* gene in *S. coelicolor*, the production of actinorhodin increased by 34 times, and the production of undecylprodigiosin increased by nine times. Researchers believe that this was caused by Lon protease producing more antibiotic biosynthetic precursors through direct protein degradation (Figure 1D) [23].

## 3. ClpP

**Figure 2:**
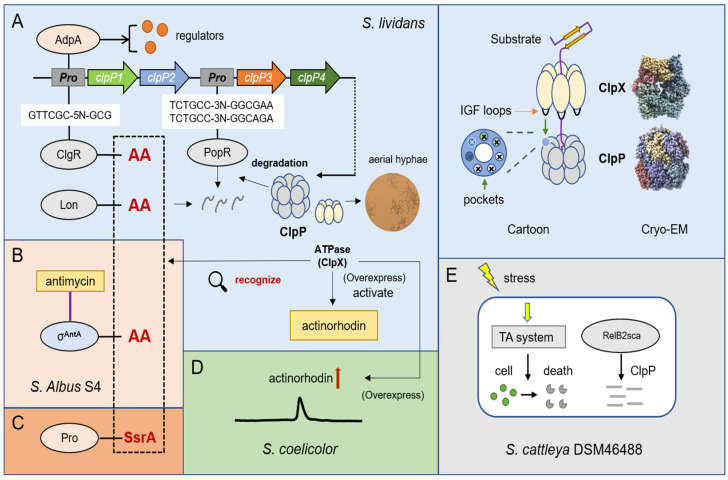
Overview of the degradation mechanism of ClpP protease and its regulation on metabolism. (**A**) Cryo—EM structures and cartoon structures of ClpP protease and ClpX (Cryo—EM structures of *E*. *coli* were downloaded from PDB database). ClpP protease can degrade important regulators ClgR, Lon, and PopR in *S. lividans*. (**B**) Two alanines in the C-terminal of σ^AntA^ are essential for degradation by ClpP in *S. albus* S4. (**C**) ClpXP can specifically degrade SsrA-tagged proteins. (**D**) Overexpression of *clpX* can increase the production of actinorhodin in *S. coelicolor*. (**E**) The type II toxin—antitoxin (TA) system is regulated by ClpP protease in *S. cattleya* DSM46488 under osmotic pressure.

Casein hydrolase ClpP is a serine protease found in the bacterial kingdom. ClpP protease is a stable heptamer ring, and each subunit has a hydrolytic active site (Ser-His-Asp) called a catalytic triad [24]. Some bacteria have a single *clpP* gene, such as *E. coli* and *Staphylococcus aureus*. However, many pathogenic bacteria and actinomycetes encode two *clpP* alleles, including *clpP1* and *clpP2* [25,26], and some even contain *clpP3*, *clpP4*, and *clpP5* [27]. There is a large family of Clp ATPases with different types of protein. The first type includes huge proteins with two ATP binding domains (ClpA, ClpB, ClpC, ClpD, and ClpE), and the second type includes smaller proteins with only one ATP binding domain (ClpM, ClpN, ClpX, and ClpY) [28,29,30]. ClpP alone can only degrade short-chain peptides in vitro [31]. If larger substrates need to be degraded, cohort regulatory ATPases (ClpA, ClpC, ClpX) are needed to promote activation. Among these substrates, research on ClpX is more extensive, because ClpXP is one of the most common proteolytic complexes, found in actinomycetes and Nitrospira bacteria, and its structure is highly conserved (Figure 2A). ClpX has asymmetric hexameric rings, which can bind to the symmetric heptameric rings of ClpP and facilitate the transfer of the substrate to the protease chamber to complete the cleavage [32]. According to reams of research, ClpX contains an important IGF ring structure (Ile268-Gly269-Phe270) in *E. coli*, which is responsible for the pocket interaction between the interface and ClpP subunit [33]. The mutations of I268L, F270L, and V274A cause severe ClpP binding defects [34].

ClpP protease has various regulatory forms in *Streptomyces*. The absence of *clpP1* and *clpP2* produces a bald phenotype in *S. lividans* [26], which implies that some undegraded proteins affect aerial hyphal growth. Additionally, the promoters of *clpP1* and *clpP2* are controlled by ClgR (a transcriptional activator), which is also confirmed in *Bifidobacterium BreveUCC2003* [35]. ClgR binds to the promoter of *clpP1* by recognizing the motif of GTTCGC-5N-GCG and can affect the expression of both *clpC* and *lon* in *S. lividans* [36]. Except for the two alleles of *clpP1* and *clpP2*, the ClpP protease of *S. lividans* also includes *clpP3* and *clpP4*, whose operons bind to PopR, a ClgR paralog. PopR activates the expression of *clpP3* operon by binding a special region: TCTGCC-3N-GGCGAATCTGCC-3N-GGCAGA [37]. Intriguingly, *clpP1* and *clpP2* are subunits mainly responsible for the degradation of ClgR, PopR, Lon, and ClpC. In the degradation test of ClpP1, ClpP2, and their substrates, some researchers found that two alanines at the end of the carboxyl were very likely to form the major site for degradation recognition, and after the alanines of ClgR-AA and Lon-AA were mutated to aspartate acid (D), the abundance of protein in cells significantly increased (Figure 2A) [38,39]. Furthermore, other researchers confirmed that a special extracytoplasmic function (ECF) RNA polymerase sigma (σ) factor σ^AntA^ in *Streptomyces albus* S4, which regulates the production of antimycin, could also be degraded by ClpXP protease and the C-terminus contains these double alanines (Figure 2B) [40]. In addition, ClpXP can specifically degrade SsrA-tagged proteins (Figure 2C). Bacteria contain a so-called trans-translation system, which is based on a bifunctional translation messenger RNA, used to rescue stalled ribosomes. The incompletely synthesized peptides are marked as C-terminal degradation signals (−AAXXXXXALAA), which is the “SsrA label” [41,42,43]. For example, the SigT protein involved in morphological differentiation and antibiotic synthesis was also degraded by ClpP/SsrA in *S. coelicolor* [44]. Combined with previous studies and reviews [45,46,47,48], there is no doubt that the substrate recognition of ClpXP mainly depends on ClpX. Additionally, in the degradation examples of *Streptomyces*, the recognition tags are actually very similar to those identified from proteomic experiments in *E. coli*. We listed five distinct identification tags, which show that ClpX is sensitive to double alanines and prefers positively charged amino acids, such as lysine, arginine, and histidine. This knowledge should prove helpful in further research on *Streptomyces* (Table 2).

AdpA, as the central regulatory protein of *Streptomyces*, not only affects mycelial growth and spore germination but also plays an important role in the biosynthesis of secondary metabolites, such as streptomycin, daptomycin, and clavulanic acid *Streptomyces griseus*, *Streptomyces roseosporus* and *Streptomyces clavuligerus*, respectively [49,50,51]. Studies confirmed that AdpA can also bind to the *clpP1* and *clpP2* operons in *S. lividans* (Figure 2A), and the lack of ClpP peptidase activity in the cell will decreases the number of AdpA, implying that they are involved in complex transcription and posttranscriptional interactions [52,53]. In addition, the overexpression of ClpX, an activator of ClpP, is able to activate the production of actinorhodin in *S. lividans* and increase the yield of actinorhodin in *S. coelicolor* (Figure 2D) [26]. The toxin—antitoxin (TA) system is a magic weapon used by bacteria to maintain an equilibrium state. It is well known that, under stress conditions such as nutrient deprivation, bacteria can degrade antitoxin proteins to produce more free toxin proteins that cause a programmed death. Some researchers first discovered the RelBE family protein of the type II toxin–antitoxin (TA) system in *Streptomyces cattleya* DSM46488, and the antitoxin protein RelB2sca can be degraded by ClpP protease to realize the response to the environment under osmotic pressure (Figure 2E) [54].

ClpP protease is also a potential target of antibacterial drugs. In *S. coelicolor*, it was found that *β*-lactones have a lethal effect on strains lacking *clpP3* and *clpP4* genes. The mechanism of action has been proposed in previous studies of *Mycobacterium tuberculosis*. It has been suggested that an inhibitory effect can be made by forming a covalent bond between the serine active site of ClpP2 and *β*-lactones. Nevertheless, it needs to be further confirmed [55].

## 4. FtsH

**Figure 3:**
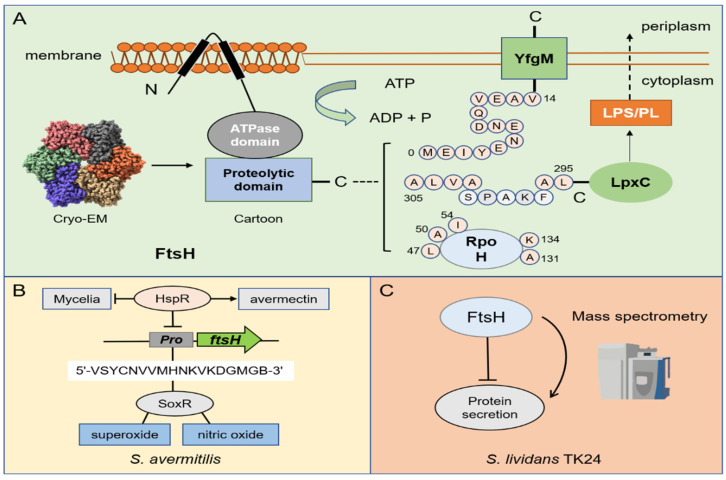
Overview of the degradation mechanism of FtsH protease and its regulation on metabolism. (**A**) The cartoon structure of FtsH protease and its Cryo—EM structure of the cytoplasmic domain from *E*. *coli* (The Cryo—EM structure was downloaded from PDB database). (**B**) Three kinds of substrates (RpoH, YfgM, and LpxC) of FtsH protease. The pink sphere represents the key amino acid in the degradation process. (**B**) In *S. avermitilis*, HspR can bind to *ftsHp* to inhibit FtsH and negatively regulate its development. SoxR can specifically bind to *ftsHp* to mediate the resistance of superoxide and nitric oxide by recognizing 18-nt binding site (5′-VSYCNVVMHNKVKDGMGB-3′). (**C**) FtsH negatively regulates the protein secretion in *S. lividans* TK24.

FtsH is the only membrane-anchored metalloprotease among the AAA+ proteases. FtsH protease is composed of three parts: the N-terminus (which contains two transmembrane regions), the AAA domain, and the C-terminus. Its molecular architecture consists of two rings where the protease domains possess an all-helical fold and form a flat hexagon that is covered by a toroid built by the AAA domains. The center of the complex hole is the active site for protease hydrolysis [56,57,58]. When ATP is supplied, FtsH is able to bind to Zn^2+^ and function on the cell membrane. The conserved HEXXH motif (X = any amino acid) in the protease domain is essential for the coordinated catalysis of Zn^2+^ [58]. Studies in *E. coli* have shown that the most important function of FtsH is to regulate the optimal ratio between phospholipids (PL) and lipopolysaccharides (LPS) on the outer membrane by degrading the key enzyme LpxC in LPS biosynthesis [59]. The degradation of LpxC is related to its C-terminal sequence and length. Concretely, the C-terminus of degron contains the sequence LAXXXXXXAVLA (X = any amino acid), and the unstructured C-terminal tail must be at least 20 amino acids long, which is necessary for FtsH-mediated degradation [60,61]. The FtsH consensus sequence for substrate degradation is inconclusive for the other two well-known substrates: YfgM (a mediator of the cytoplasmic and extracytoplasmic stress responses) and RpoH (the master regulator of the heat-shock response). The degradation process of YfgM no longer requires the C-terminal sequence, but the first 14 amino acids (MEIYENENDQVEAV-) of the cytoplasmic N-terminal sequence [62]. Notably, label-free quantitative proteomics excavated similar motifs in *E. coli*, and it can be speculated that FtsH may be sensitive to glutamic acid and aspartic acid (Table 2) [63]. However, the degradation of RpoH depends on several scattered amino acids. The amino acid substitutions of L47, A50, and I54 affect the hydrolysis of RpoH [64,65,66], and the replacement of A131, and K134 influences the stability of RpoH itself (Figure 3A) [67].

FtsH is relatively less studied in *Streptomyces* compared with other proteases, so its molecular mechanism is not very clear in the regulation of *Streptomyces*. SoxR, which is a redox-sensitive transcriptional regulator, has been shown to mediate the resistance of superoxide and nitric oxide under oxidative stress in *E. coli* [68]. By comparing its target homologous gene of *S. coelicolor* with the *ave* gene in *S. avermitilis*, researchers predicted the 18-nt SoxR binding site (5′-VSYCNVVMHNKVKDGMGB-3′) in their promoter region (V  =  A/T/C; S  =  C/G; Y  =  T/C; N  =  A/G/C/T; M  =  A/C; H  =  A/T/C; K  =  G/T; B  =  G/C/T). Surprisingly, it is found that *ftsHp* meets this sequence feature and can specifically bind to SoxR. Researchers believe that although FtsH is not a key developmental gene of *Streptomyces*, it has a certain degree of effect on cell differentiation [69]. Later, studies on the heat shock-related inhibitor HspR in *S. avermitilis* found that HspR can bind to *ftsHp* to directly inhibit FtsH and negatively regulate its development. Nevertheless, this regulatory process is beneficial for the production of avermectin (Figure 3B) [70]. With the development of multiomics in recent years, some research groups have used transcriptomics and proteomics to explore the biological functions of FtsH protease in *S. lividans*. Seventeen proteins encoded by protease and protease inhibitor genes with a significant abundance have attracted attention with the change in the growth stage. When FtsH is deleted, the protein secretion capacity of *S. lividans* TK24 was significantly improved (Figure 3C). In comparison to other mutant strains, TK24*ΔftsH* also showed the most severe phenotypic changes. Therefore, FtsH is considered to be a major global regulator in *S. lividans* [71]. Similar research on FtsH was also conducted for other microorganisms. For example, the lack of FtsH strongly increases the abundance of ten cytoplasmic and membrane proteins in *Corynebacterium glutamicum* without affecting its growth [72]. Since secretion is a transmembrane cross process and FtsH functions on the cell membrane, we can speculate that *Streptomyces* is likely to be indirectly regulated by FtsH in the efflux and transport of antibiotics. Although it is greatly challenging to conduct research on membrane proteins, it is valuable for industrial applications.

## 5. Proteasome

**Figure 4:**
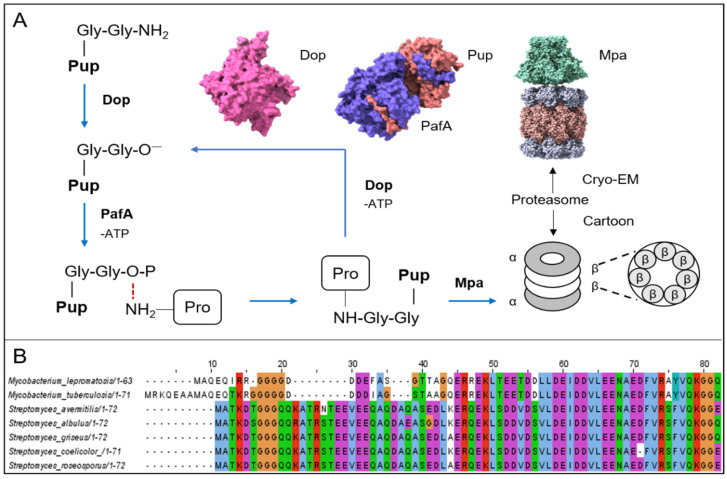
Overview of the degradation mechanism of Pup–proteasome and different types of Pup. (**A**) Degradation diagram of the interaction between the proteasome, Pup, Dop, PafA, and Mpa. The pink Cryo—EM structure of Dop is identified from *Acidothermus cellulolyticus*. The Cryo—EM structure of Pup-PafA complex is identified from *Corynebacterium glutamicum*. The purple area represents PafA, and the orange area represents Pup. The Cryo—EM structure of the Mpa—proteasome complex is identified from *Mycobacterium tuberculosis* (the Cryo—EM structures were all downloaded from the PDB database). (**B**) The alignment of Pup amino acid sequences between *M. lepromatosis*, *M. tuberculosis*, *S. avermitilis*, *S. albulus*, *S. griseus*, *S. coelicolor,* and *S. roseosporus* (amino acid sequences were downloaded from the NCBI database).

Proteasomes are ubiquitous in actinomycetes. Their structures are the same as eukaryotic homologs, consisting of 28 subunits: two homoheptamer loops of β subunits containing the active site are sandwiched between the two homoheptamer loops ofα subunit, which prevents foreign proteins from entering the cylindrical proteolytic chamber in the absence of an activator (Figure 4A) [73]. In eukaryotic cells, some proteins are labeled with ubiquitin molecules and then recognized and degraded by the proteasome. In prokaryotic cells, the proteasome was first discovered in actinomycetes in the 1980s. However, a thorough understanding of the proteasome degradation mechanism was first achieved in 2008, when Pearce et al. discovered a protein with similar functions to ubiquitin in *M. tuberculosis* and named it prokaryotic ubiquitin-like protein (Pup) [5]. As a new degradation pathway, the Pup–proteasome system is a distinctive way to regulate intracellular protein levels and eliminate misfolded proteins and participates in regulating a variety of physiological functions in cells. The sequence K/RGGQ at the C-terminus of the Pup molecule contains the conserved Gly-Gly sequence in ubiquitin, which can specifically link with Lys to form a Gly-Gly-Lys isopeptide bond [74]. Generally, the carboxyl-terminal sequence of Pup is Gly-Gly-Gln, and Gln needs to be deamidated to Glu under the action of Dop (deamidase) to form covalent bonds [75]. Taking advantage of this feature, in 2009, Festa et al., used tandem affinity chromatography and mass spectrometry to systematically analyze the Pup–labeled protein in *M. tuberculosis*, according to whether the protein’s lysine site has the matching peptides of GGE (243 Da) to determine modification by Pup (Figure 4A) [76]. Of course, there are some prokaryotic cells that have Glu at the carboxyl end of Pup (Figure 4B). For example, when recombinant Pup, PafA (Pup ligase), and Dop were purified and expressed in vitro by *Streptomyces hygroscopicus*, it was found that although Dop is redundant as a deamidase when the tail of Pup is glutamate, it still has the ability to recycle Pup (a recycler of Pup by depupylation). Finally, these authors used the molecular mass of covalent modification to identify a Pup substrate (SHJG_3659) [77]. The depupylation function of Dop has also been confirmed in other microorganisms and is not explored further in this study [78,79]. When Pup is deamidated, it will hydrolyze ATP under the action of PafA and catalyze the phosphorylation of Glu [80], forming an intermediate that is connected to the substrate Lys [81]. Then, Pup–protein will interact with Mpa (mycobacterial proteasome ATPase/the switch that regulates the “gate” of the proteasome α subunit) and be delivered to the proteasome for degradation (Figure 4A) [82,83,84,85].

The 20S proteasome is composed of two types of subunits (PrcA and PrcB) in *S. coelicolor* and has a chymotrypsin-like activity in synthetic substrates [4]. The most significant change caused by the loss of proteasome in *S. coelicolor* is the clear increase in the level of nonheme chloroperoxidase SCO0465, which is consistent with the increase in resistance to hydrogen peroxide [86]. In addition, the PafA deletion mutant has spore-producing defects, and the deletion of Pup shows greater changes than the proteasome mutants [87], including the significantly reduced production of the secondary metabolites, undecylprodigiosin and actinorhodin [88]. This implies that Pup has other functions aside from directing protein targeting proteasome degradation in *S. coelicolor*. Notably, subsequent studies in other microorganisms have verified this finding. Researchers have found that pupylation is not limited to protein degradation. In the case of oxidative stress, and carbon and nitrogen starvation, *Corynebacterium glutamicum*, which lacks proteasomes, opens up this kind of protein modification and causes the phenomenon of ferritin release [89]. One study focused on the proteasome in *S. lividans* and found that the proteasome deletion strain had no significant impact on phenotypes compared with the wild-type strains; however, a high yield can be achieved for soluble human tumor necrosis factor receptor II (shuTNFRII) and salmon calcitonin (sCT) [90]. The same situation also occurs in *S. roseosporus*. Although the deletion of the proteasome causes a bald phenotype and makes the bacteria lose the production capacity of red pigment and daptomycin, it is a better host for expressing the protein than WT [91]. This result indicated that the disruption of proteasome genes might help to efficiently produce heterologous proteins, which provides a new strategy for heterologous expression. In prokaryotes, in addition to interaction with Pup molecules, the proteasome may also interact with the Cdc48-like protein (Cpa) of actinomycetes to achieve the degradation of certain proteins. It has been proven that Cpa can interact with 20S core particles in vitro to form ring stacks and collinear complexes, but no degradation substrate has been discovered thus far [92,93]. Additionally, tunicamycin, a fermentation product of *Streptomyces hygroscopicus*, can inhibit the ubiquitin-proteasome system in eukaryotic cells rather than its own proteasome degradation system [35]. This is a vital discovery regarding the application of the proteasome, but its mechanism remains unclear. It has been a very effective drug development strategy by using ubiquitin ligase to induce ubiquitination and subsequently transport the target protein to the 26S proteasome for degradation. However, it is still limited to eukaryotes. To date, some researchers have found that the recognition ligand linked to arginine (B_3_A) and protected by tert-butyl carbamate (Boc3) can induce ligand degradation. This process requires the proteasome but does not involve the ubiquitination of the target protein. The B_3_A ligand stimulates the activity of the purified 20S proteasome, which allows the tag to directly bind to the 20S proteasome [94], providing a novel strategy for degradation in prokaryotes. Based on this, it is possible to control the metabolic pathways to benefit antibiotic production during the growth of actinomycetes, which is achieved by inducing the degradation of certain proteins.

## 6. Cross Talk

Protease and proteasome together constitute a protein quality control system in bacteria, which sometimes creates a more complex regulation network for understanding a single protein degradation process. Since they are able to function alternately, they pose a huge challenge for further research.

An early study on the protease of *E. coli* discovered that the mutagenic protein UmuD’ and its homodimers-UmuD were involved in a complex proteolytic pathway. As the precursor of UmuD’, UmuD has no mutagenic activity and can be degraded by Lon protease in vivo. However, UmuD’ is not affected by Lon protease and is degraded by the ClpXP protease [95,96]. Intriguingly, the N-terminal tail is proven to be the key recognition site for UmuD to participate in the degradation of Lon, and UmuD’ is also targeted to ClpXP through the 24 amino acids of the N-terminus of UmuD, which is specifically degraded [97]. In addition to *E. coli*, examples of ClpXP and Lon synergistic action are also found in *Caulobacter crescentus*. CtrA, a positive global regulator, promotes the expression of DNA methyltransferase CcrM to complete cell division. *ccrM* transcription stops just before cell division when the CtrA is degraded by ClpXP. Soon after, CcrM is continuously degraded by Lon [98,99].

In *S. coelicolor*, the SigT protein responsible for the negative regulation of morphological differentiation is in a stable state under the binding of anti-σ factor RstA, which protects itself from independent proteasome degradation. However, if RstA is degraded, the exposed SigT will bind to the promoter regions of *clpP1* and *clpP2* and induce the production of ClpP protein so that it can be degraded itself. Surprisingly, undecylprodigiosin and actinorhodin can regulate the binding of SigT and *clpP1/2* in this degradation process. The dual regulation of ClpP and proteasome indicates that it has a complex and delicate degradation system in *S. coelicolor.* In addition, after the overexpression of the two pathway-specific genes *redD* and *actII-orf4* in the proteasome-deficient strain, compared with the original strain, it increased the yield of undecylprodigiosin by 3–5 times, and the yield of actinorhodin by 9–30 times, indicating that the production of antibiotics is closely related to the degradation system [44,100].

*S. roseosporus* is the producer of daptomycin. Due to the special feature of its precursor, adding capric acid during fermentation is considered a necessary means to increase its production. Unfortunately, capric acid (>2.5 mM) is toxic to *S. roseosporus*. Under this kind of exogenous stimulation, transcriptomics surprisingly found that the genes encoding proteasome and ClpB protease were significantly upregulated, responding to oxidative stress by degrading misfolded proteins [101]. Unfortunately, there is a lack of research that explicitly focuses on this mechanism, but it provides the basis to address the toxicity of decanoic acid from the perspective of protein degradation.

## 7. Conclusions

Despite the progress of protein degradation thus far, our knowledge of microbial proteostasis remains quite limited, the association between protein degradation and the primary/secondary metabolism or morphological differentiation is undeniable, yet more investigations are still essential. In particular, the following areas warrant a thorough investigation. (i) Degradation mechanisms need to be dissected, especially the featured degradation signals of target proteins. Although some degradation signals, such as the N-terminus for Lon and FtsH, SsrA tag for ClpXP are important, the key amino acid residues or conserved motifs and characteristic features seem to be unclear. Furthermore, how the signals are accurately recognized and the time when the degradation of target proteins is switched on and off, need to be resolved. (ii) The regulatory mechanism of protein degradation must be considered. In a few cases, we know that some regulators, such as ClgR for ClpP and HspR for FtsH, are involved in the regulation of protein degradation. However, generally, the cascade regulatory network is complex, thus the regulatory pathways of protein degradation remain indistinct. The many global or pleiotropic regulators that participate in proteostasis and how the consortium of regulators work together to affect protein synthesis and degradation must be systematically revealed. Considering the complexity of regulation, integrating multi-omics, such as transcriptomics, proteomics, and even translatomics would be an efficient strategy to comb the regulatory network of proteostasis. (iii) The temporal regulation of protein degradation is pivotal for the practical applications of proteostasis for improving the performance of microbial cells and enhancing the production of value-added compounds, such as polypeptides, antibiotics, or enzymes. The abundance of proteins, particularly key or rate-limiting enzymes, is closely related to the biosynthesis of target compounds. Proteases serve important functions in protein quality control, which is also a dynamic process. Some studies suggest that engineering protease-targeted proteins can stabilize them against degradation. More importantly, the development of synthetic biology endows us with the ability to engineer controllable protein degradation. Therefore, based on the degradation and regulatory mechanisms of proteostasis, we can perform the dynamic quality control of target proteins by regulating proteases or the modification of target proteins for facilitating the production of target products. Here, we emphasized the importance of *Streptomyces* in microbial drugs and summarized the development of protein degradation and antibiotic biosynthesis. In *Streptomyces*, the time-course metabolic shift from primary to secondary metabolism especially requires the temporal regulation of different proteins, which assist with the growth in the growth stage and secondary metabolism in the stationary stage. In particular, the stability and availability of the enzymes directly involved in antibiotic biosynthesis in the stationary stage are very important for drug production. Apart from overexpression, genetic modification or the fine-tuning of protein degradation are alternative methods for stabilizing these against degradation. (iv) Proteostasis advances the development of cell-free protein synthesis systems. Based on the extended knowledge of protein degradation, we can develop more efficient cell-free systems by engineering protease-deficient hosts or stabilizing target proteins. In *E. coli*, protease-deficient mutants were constructed to reduce protein degradation and used for cell-free protein synthesis. Although many *Streptomyces*-based cell-free protein synthesis systems were developed and showed a considerable advantage, a large number of proteases in *Streptomyces* limit their extensive applications and the expensive addition of a protease inhibitor is usually required. In future research, we can thoroughly explore protein degradation and delete all active proteases in *Streptomyces* to develop highly efficient cell-free protein synthesis systems.

In summary, due to the robustness of bacteria, numerous endogenous proteins are degraded by proteases or proteasomes and cannot be conveniently captured or studied. Therefore, understanding the process of protein degradation is an indispensable part of analyzing the complex regulatory network and performing rational strain engineering to improve the production of value-added compounds in microorganisms. Continually revealing the interaction of proteases or proteasomes with other regulators not only helps medicinal bacteria, such as *Streptomyces* to serve better as producers of antibiotics, but it may also provide a new theoretical basis for research on other pathogens.

## Figures and Tables

**Table 1:** Summary of sources and functions of twelve compounds.

Compounds	Source	Function
Daptomycin	*Streptomyces roseosporus*	Antibiotics (Gram-positive infections)
Rapamycin	*Streptomyces rapamycinicus*	Immunosuppressants/Antifungal agents
Amphotericin B	*Streptomyces nodosus*	Antifungal drugs
Adriamycin	*Streptomyces peucetius*	Anticancer drugs
Ivermectin	*Streptomyces avermitilis*	Antiparasitic drugs
Ectoine	*Halomonas elongate*	Bioprotection agents
Pyoluteorin	*Pseudomonas fluorescens* Pf-5	Antibiotics/Antifungal agents
Actinorhodin	*Streptomyces coelicolor*	Antibiotics (redox-active)
Undecylprodigiosin	*Streptomyces coelicolor*	Antifungal/Antitumor agents (Potential)
Clavulanic acid	*Streptomyces clavuligerus*	β-lactamase inhibitor
Streptomycin	*Streptomyces griseus*	Antibiotics (Tuberculosis)
Tunicamycin	*Streptomyces hygroscopicus*	Antibiotics/Antifungal agents

References are listed in the corresponding sections.

**Table 2:** Five distinct tags of ClpXP degradation substrate and similar motifs of FtsH degradation substrate identified from *E. coli* by proteomic experiments.

ClpXP Substrate		
Location	Protein Name	Sequence
**C-motif-1**	SsrA	LAA
	YdaM	LAA
	LldD	NAA
	Gcp	PAA
	RpIJ	EAA
**C-motif-2**	MuA	RRKKAI
	YbaQ	RAKKVA
	PncB	HIKKAS
	Rsd	RVKHPA
	PaaA	HARKVA
**N-motif-1**	AtpD	ATGKI-
	Dps	STAKL-
	GapA	TIKV-
	**λO**	TNTAKI-
	σ^S^	SQNTLKV-
**N-motif-2**	DadA	MRVVI-5-V-
	FabB	MKRAV-5-I-
	IscR	MRLTS-5-V-
	IscS	MKLPI-5-A-
	OmpA	MKKTA-5-V-
**N-motif-3**	Crl	TLPSGHPK-
	DksA	MQEGQNR-
	GlcB	SQTITQSRLR-
	KatE	MSQHNEK-
**FtsH substrate**		
**Location**	**Protein name**	**Sequence**
**N-motif**	YfgM	MEIYENENDQVEAV
	ExbD	MAMHLNENLDDNGEMH
	YlaC	MTEIQRLLTETIESL

References are listed in the corresponding sections.

## Data Availability

Not applicable.
